# Cognitive Interaction Technology in Sport—Improving Performance by Individualized Diagnostics and Error Prediction

**DOI:** 10.3389/fpsyg.2020.597913

**Published:** 2020-12-21

**Authors:** Benjamin Strenge, Dirk Koester, Thomas Schack

**Affiliations:** ^1^Neurocognition and Action Group, Faculty of Psychology and Sports Science, Center for Cognitive Interaction Technology (CITEC), Bielefeld University, Bielefeld, Germany; ^2^Sport Psychology, Faculty Business and Management, BSP Business School Berlin, Berlin, Germany

**Keywords:** karate athletes/performance, karate kata, SDA-M, ethical issue recognition, mental representation structures, cognitive assistance systems

## Abstract

The interdisciplinary research area Cognitive Interaction Technology (CIT) aims to understand and support interactions between human users and other elements of socio-technical systems. Important reasons for the new interest in understanding CIT in sport psychology are the impressive development of cognitive robotics and advanced technologies such as virtual or augmented reality systems, cognitive glasses or neurotechnology settings. The present article outlines this area of research, addresses ethical issues, and presents an empirical study in the context of a new measurement and assessment system for training in karate. Recent advances in the field of cognitive assistance systems enabled largely automatized assessments of individual mental representation structures for action sequences, such as choreographed movement patterns in dance or martial arts. Empirical investigations with karate practitioners of different skill levels demonstrate that advanced software-based survey and algorithmic analysis procedures based on cognitive models generate individualized performance predictions for a movement sequence from the *Kanku-dai kata* (a pre-defined karate movement sequence), which correlated significantly not only with formal expertise (*kyu/dan* rank) but also with the actual likelihood of mistakes in action execution. This information could prospectively be used to define individual training goals for deliberate practice and incorporated into cognitive interaction technology to provide appropriate feedback. We argue that the development of cognitive interaction systems for sport should explicitly take ethical issues into consideration and present a particular developed engineering approach. The potential benefits of such an assistance system for intermediate and advanced practitioners include more effective and flexible practice, as well as supportive effects, and more flexible training schedules. Furthermore, we argue that researchers from the field of sport psychology can benefit from advances in technological systems that enhance the understanding of mental and motor control in skilled voluntary action.

## 1. Introduction

For over a decade numerous researchers from psychology, computer science, engineering, biology, linguistics, and sports science shaped the interdisciplinary field of Cognitive Interaction Technology (CIT) in order to establish the scientific and technological basics for creating systems that are capable of interacting at different levels of cognitive complexity (Ritter and Sagerer, 2009). Pursuing the vision of intuitive, human-friendly technology that adapts to users' needs (Wachsmuth et al., 2012) by offering intuitive and personalized support in daily routines (Wrede et al., 2017), CIT comprises research topics such as motion intelligence, attentive systems, situated communication, memory and learning (Wachsmuth, 2008; Schack and Ritter, 2013). A major goal is “*to develop memory systems that can approximate some of the key features of human memory, such as flexible association, scalability and learning at different levels”* (Ritter, 2010, p. 230). While classic artificial intelligence concentrates on modeling the mind, CIT research focuses more on interactions that take place in the physical world (Wachsmuth et al., 2012) and combines algorithmic approaches with insights from analyses of human and animal motion to establish “a coherent picture about the internal representation of our movement abilities” (Ritter, 2010, p. 230). On the technical side, CIT combines visualization, sonification, haptic, and augmented reality devices, motion capture, simulated agents in virtual worlds, and attentive user interfaces in novel ways (Ritter, 2010). This led to a broad range of technological advancements such as embodied anthropomorphic robots that can aid humans (Ritter, 2010; Wachsmuth et al., 2012), intelligent glasses for cognitive assistance (Essig et al., 2016), and smart environments systems with mobile service robots for ambient assisted living (Wrede et al., 2017).

Sport psychology researchers and practitioners have been traditionally concerned with topics like analyzing and improving human performance but started to develop new technologies (e.g., Schack and Ritter, 2013) to support sport performance several years ago, e.g., using motion tracking, eye tracking, heart-rate variability or EEG measurements to provide biofeedback with sonification, virtual and augmented reality systems (see e.g., Schack et al., 2014a, 2020; Hagan et al., 2018, for overviews). A main question is how to inform assistive technologies about the cognitive background (memory) and motion intelligence (motor skills) of the user. From a traditional cognitive psychology perspective (see Anderson, 2010), the development of human expertise is commonly characterized by *proceduralization*: The learner integrates declarative knowledge into procedural rule sets so that less declarative memory needs to be used, which reduces brain activation in areas like the hippocampus, prefrontal cortex, and anterior cingulate, and decreases latency. Fitts and Posner (1967) famously described this process as a three-stage model, which transitions from an initial “cognitive stage” to an intermediate “associative stage" and terminates in the “autonomous stage.” Research has also found that, while potential performance improvements are limited by factors like musculature and age, the time required for cognitive processing may converge against zero as a power function of practice (Anderson, 2010). This characterization of human expertise development has been challenged by the sport psychological theory of *deliberate practice*, which means engaging in training that focuses on improving specific tasks and involves providing immediate feedback, time for problem-solving and evaluation, and opportunities for repeated performance in order to refine behavior (Ericsson, 2008). This obviously requires that practitioners are given specific tasks with well-defined goals (Ericsson, 2007). Purportedly, deliberate practice continually improves performance, because “*expert performers counteract automaticity by developing increasingly complex mental representations to attain higher levels of control of their performance and will therefore remain within the cognitive and associative phases”* (Ericsson, 2008, p. 991).

Based on a Cognitive Action Architecture Approach (CAA-A), sport psychology researchers described the building blocks and levels of the action system that enable us to control movements such as striking the tennis ball at the right time, or coordinating steps and arm movements in dancing or golf, and demonstrated how the measurement of mental representation can be used for applied work in sport, new pathways in mental training (imagery), and to inform technical systems (Tenenbaum et al., 2009; Frank et al., 2014; Schack, 2020). A highly promising application of interactive technology in sport psychology is to provide helpful assistance to athletes in the context of learning. In coaching, trainees' capabilities to respond to an expert's assistance and the coaching system's ability to activate users' learning potential can be observed (Schack, 2020). Coaching a trainee at different interaction levels while practicing and learning a motor task constitutes an interesting scenario not only for supporting motor learning processes but also to understand the effectiveness of current coaching principles (see also Schack, 2020). Based on mental representation analyses in sport (Schack and Mechsner, 2006; Schack and Hackfort, 2007; Schack, 2020), we investigate how coaching could become more individualized and adaptive in the real world and in Virtual or Augmented Reality settings (Schack et al., 2020). To this extent, it is clearly advantageous for a real or virtual coach to know how mental structures form, stabilize, and change in sports (Schack, 2020). Coaches who possesses such knowledge are better able to address the individual athlete on his or her current level of learning and shape instructions to improve training and performance (Schack, 2020).

In this line of research, numerous studies found that the differing mental representation structures of experts and novices can be measured with the “*structural-dimensional analysis of mental representations”* (SDA-M) method (Schack, 2012) and influenced by appropriate training (e.g., Heinen et al., 2002; Schack, 2004; Schack and Mechsner, 2006; Schack and Hackfort, 2007; Frank et al., 2013, 2014; Schack et al., 2014b). A methodological review and evaluation of research in expert performance in sport by Hodges et al. (2007, p. 164) noted that the SDA-M method “*is expected to aid in our understanding of the usually non-declarative motor representations underlying expert performance in fast, complex coordinative actions and in identifying the problems novices encounter in understanding motor problems.”* Recently, Strenge et al. (2019) described advanced algorithms for automatized analyses of task-related mental representation structures based on SDA-M related to action sequences. These algorithmic approaches might be useful as a component of future CIT systems, like cognitive glasses, to measure and improve human performance in sport. In this context, SDA-M and its recent algorithmic extensions could serve as a measurement and assessment tool, and smart glasses or other portable devices could provide corresponding feedback for deliberate practice.

The present study reports on an empiric study in karate as a proof of concept for this assessment approach. To this end, the article first recapitulates the SDA-M method and its algorithmic extensions and then describes the specific study-related methods. Subsequently, potential ethical benefits and risks, as well as links to ethical aspects of technical system development, are discussed.

## 2. Retrieval and Analysis of Mental Representation Structures with SDA-M

The SDA-M method can be used to analyze human memory structures with respect to a specified set of items (e.g., basic actions in sports). SDA-M consists of several survey and analysis steps, which are briefly outlined in the following. The theoretical, methodical and algorithmic foundations of SDA-M have been described in detail by Schack (2012), and Strenge et al. (2019) presented and exemplified recent algorithmic extensions for automatic assessment of SDA-M data concerning individual likelihoods of errors in action sequences.

### 2.1. Task Analysis

In a preparatory step, it is generally important to understand the motor task (here: a karate movement sequence) and characterize its task-adequate functional organization, e.g., in cooperation with athletes of different levels of expertise or coaches. The activity is hereby split into “basic action concepts” (BACs, see Schack, 2012), which are represented by textual descriptions and/or images. This can be done by researchers with the help of a functional movement analysis (Hossner et al., 2015) and together with domain experts to establish a plausible and workable set of BACs.

### 2.2. Step A: Split Procedure and Distance Scaling

**Figure 1:**
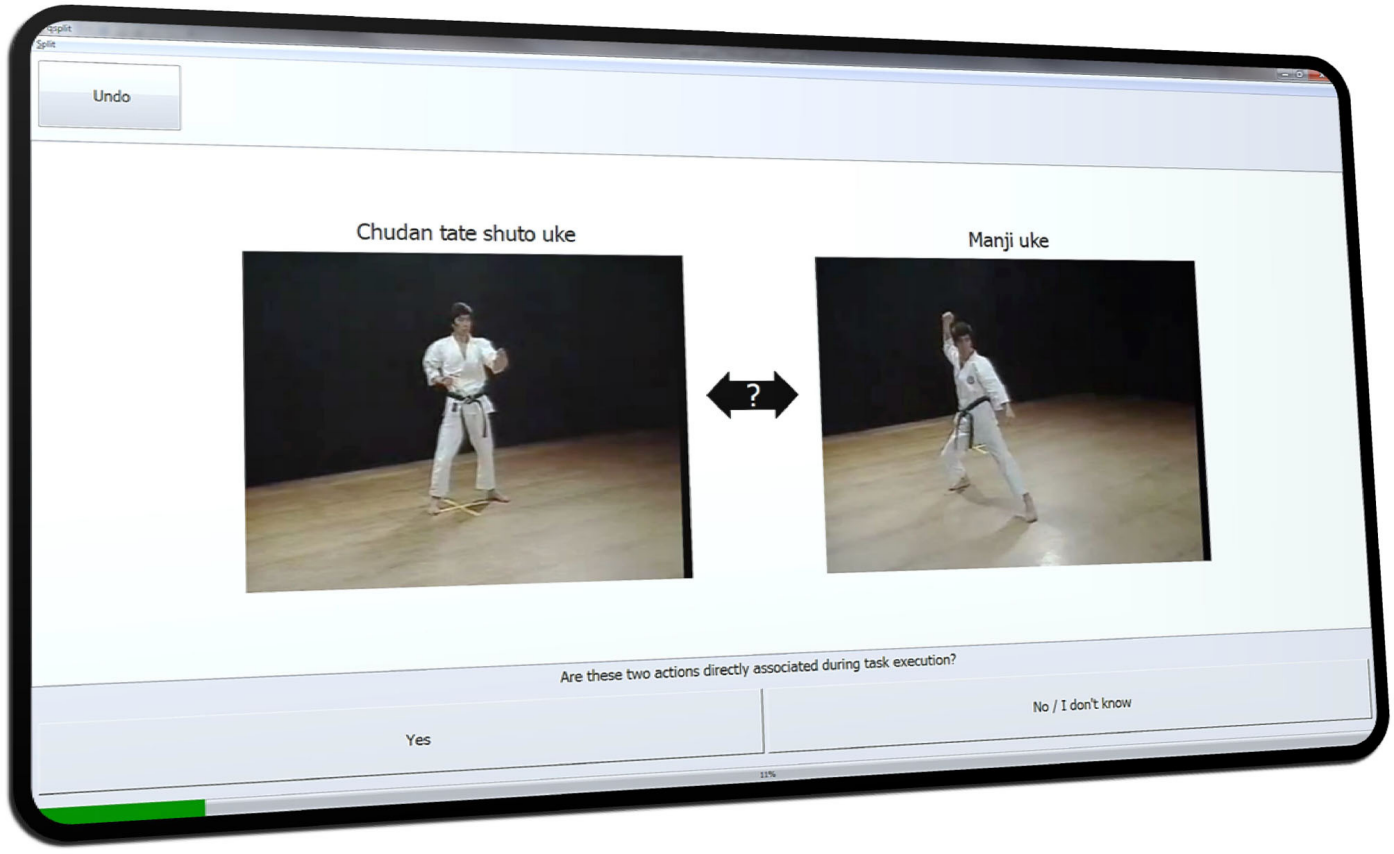
User interface of the QSplit software for the SDA-M split procedure showing two basic actions (karate blocking techniques) from the *Kanku-dai* movement sequence.

During the split procedure these action items (BACs) are shown to study participants on a screen using specialized software such as the *QSplit* SDA-M tool (see Figure 1). The *n* actions of the analyzed task or activity are presented in random order as reference objects or “targets,” and all *n* − 1 other actions are then compared to the current target (also in random order). For each pair of actions the participant must decide whether or not these are directly associated during execution of the analyzed activity (e.g., a movement sequence). The SDA-M software then calculates correlation and distance values between all pairs of actions.

### 2.3. Step B: Hierarchical Clustering and Visualization

**Figure 2:**
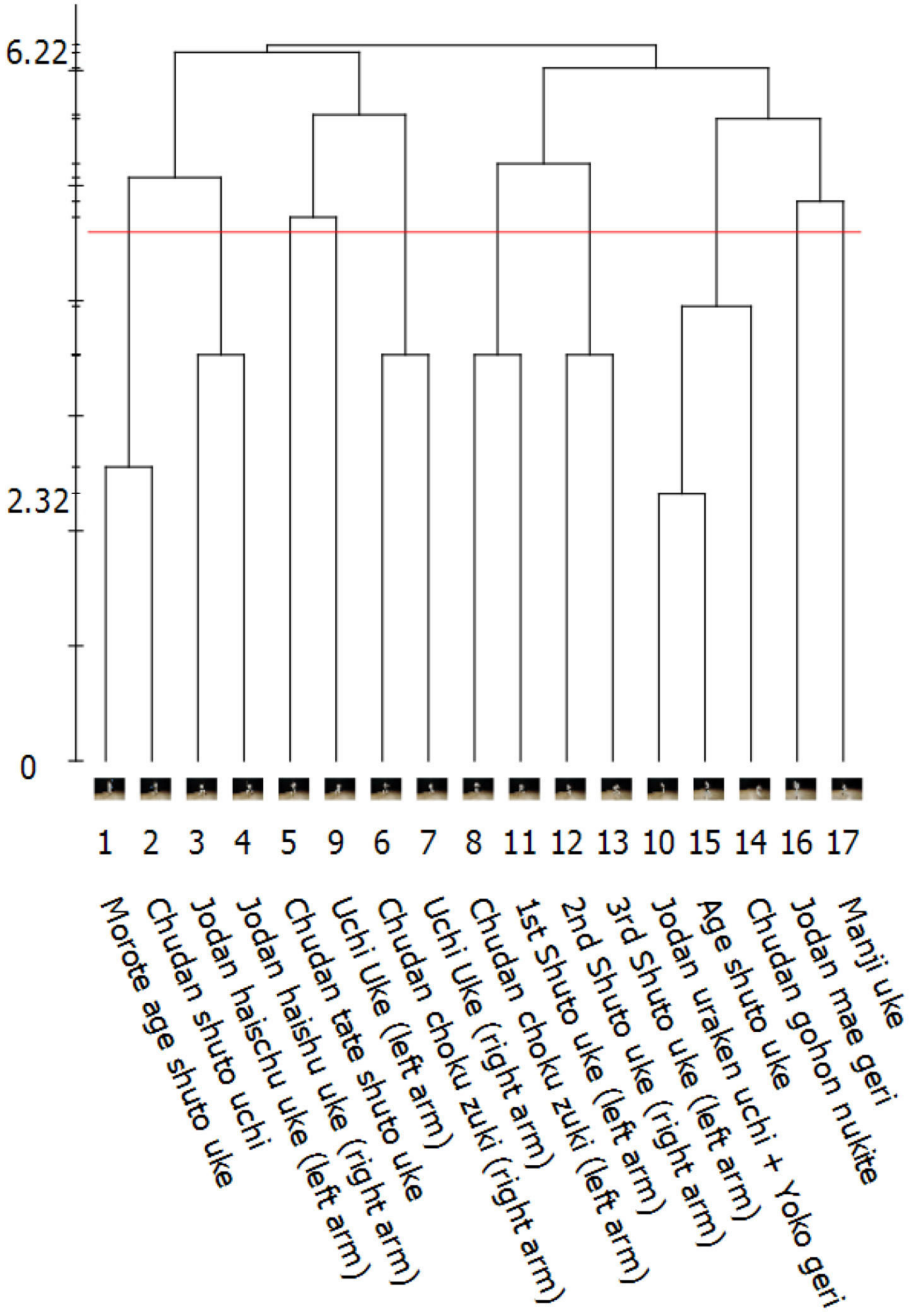
Visualization of a 5th *kyu* (blue belt) karate practitioner's individual mental representation structure related to the *Kanku-dai* movement sequence by an SDA-M dendrogram. Numbers below the dendrogram indicate designated positions of each action within the sequence.

**Figure 3:**
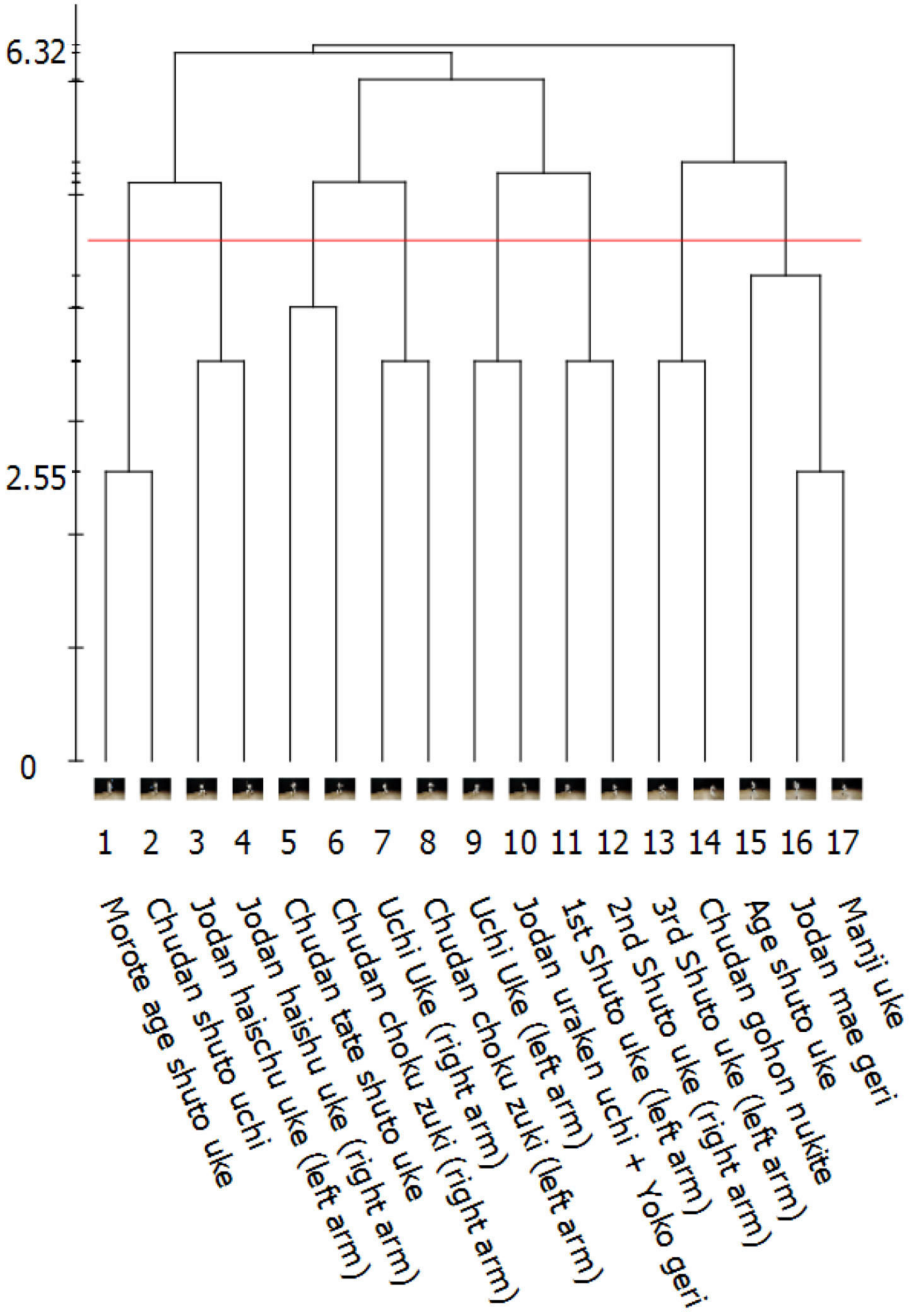
Visualization of a 2nd *dan* (black belt) karate practitioner's individual mental representation structure related to the *Kanku-dai* movement sequence by an SDA-M dendrogram. Numbers below the dendrogram indicate designated positions of each action within the sequence.

The results from step A can be used to create a hierarchical agglomerative average-linkage clustering of the actions (BACs). The SDA-M software visualizes this clustering with a dendrogram to enable manual assessment of participants' mental representation structures (see Figures 2, 3 for examples from the present study). For many SDA-M applications this is the last necessary analysis step (see e.g., Heinen and Schwaiger, 2002; Heinen et al., 2002; Heinen and Schack, 2004; Schack, 2004; Schack and Hackfort, 2007). Further steps like investigating the feature dimensions of the representation or invariance measures are possible (Schack, 2012).

### 2.4. Step C: Automatized Algorithmic Assessment of SDA-M Data

Two different algorithmic approaches for predicting human error based on SDA-M data have been developed and presented by Strenge et al. (2019): *Analysis of Most Probable Actions* (AMPA) and *Correct Action Selection Probability Analysis* (CASPA). These new algorithmic approaches automatized the process of assessing memory structures based on SDA-M data to predict probable errors in action sequences, which eliminated the previous need for manual assessments using dendrograms.

The basic approach of AMPA is to determine if the set of actions that have lowest distance to the previously executed action, which corresponds to strongest association, contains a correct follow-up action. This results in a simple binary assessment ∈ {0, 1} for each action *a*_*i*_ with *i* ∈ {1, ..., *n* − 1} that indicates whether participants would be able to select a correct action for the next step or not. The more sophisticated CASPA algorithm is based on parts of the computational cognitive architecture “Adaptive control of thought–rational” (ACT-R) (Anderson and Lebiere, 1998; Anderson et al., 2004). CASPA uses the same individual SDA-M data as AMPA but outputs a continuous measure *p*_*i*_ ∈ [0, 1] to estimate the probability of correct action selection after action *a*_*i*_. Arbitrary thresholds for *p*_*i*_ can be used to decide if assistance will be needed. This binarized output of CASPA is referred to as CASPA_*d*_ when using a default threshold of 0.5, whereas usage of an empirically informed task-specific threshold is denoted as CASPA_*i*_.

Strenge et al. (2019) speculated that the results of AMPA or CASPA could be used by technical systems like intelligent glasses to provide anticipatory action support.

## 3. Methods

**Figure 4:**
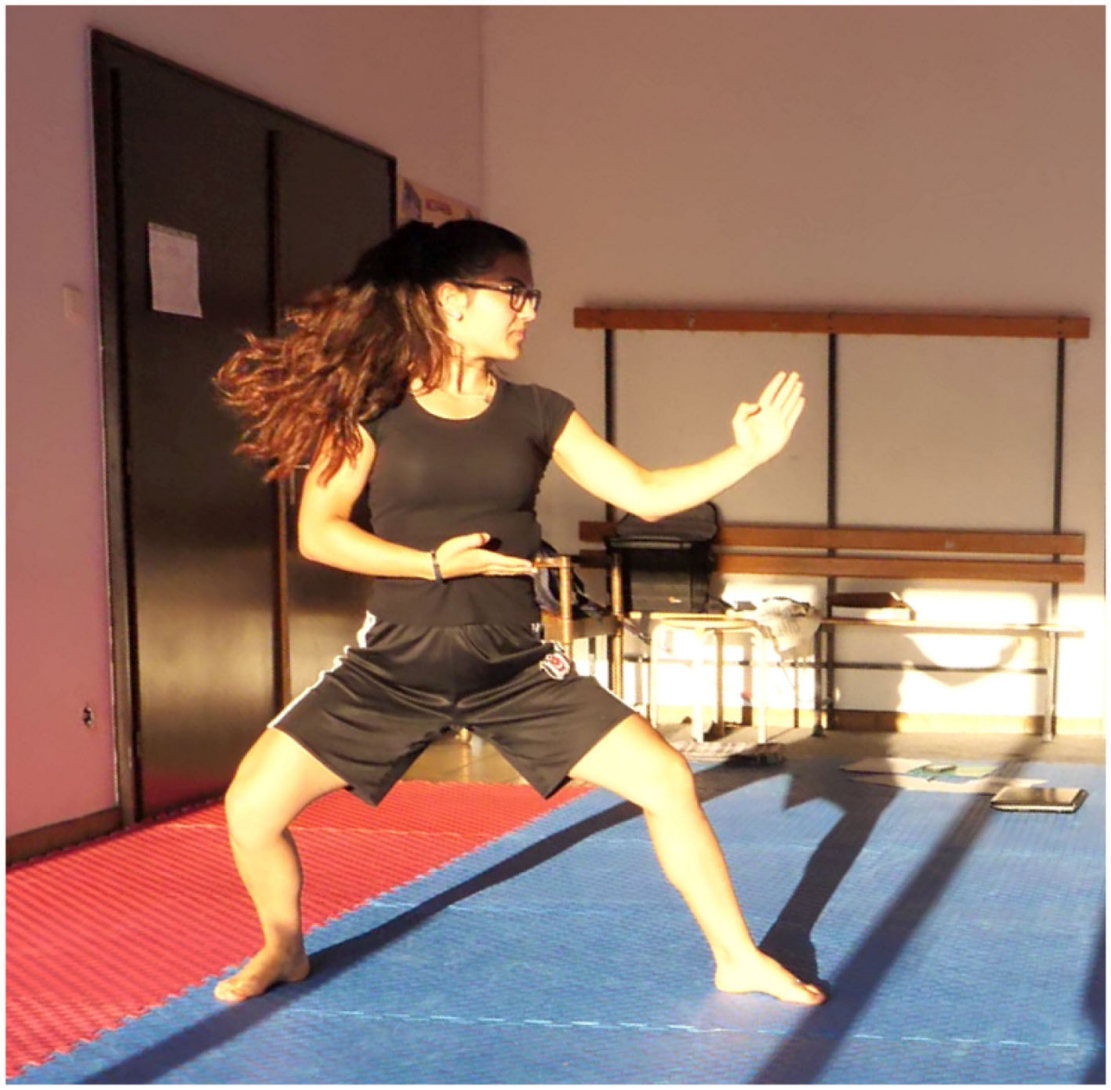
Participant executing a *shuto uke* (“knife hand block”) karate technique from the *kanku-dai kata* movement sequence.

Karate practitioners of different skill levels were analyzed regarding a choreographed sequence of distinct movements (karate techniques) from the beginning of the so-called *Kanku-dai kata*. These karate techniques include blocks and strikes using one's hands or feet, and occasionally both at the same time, delivered from specific stances. While executing a *kata* movement sequence, technique executions may be accompanied by hip rotations and/or a transition to another stance, thus effectively making them full-body movements despite the fact that they are commonly denoted just by the type of block or strike. For example, each successive *shuto uke* (“knife hand block”) in the *Kanku-dai* sequence implicitly includes moving one step forward in a *kokutsu dachi* (a defensive back stance where most body weight rests on the rear leg) switching the front and rear leg on each step (see Figure 4). Instructors of the popular *Shotokan* style of karate commonly introduce the *Kanku-dai* at some point during students' preparation for the first *dan* black belt or “master” level. The *Kanku-dai kata* can be understood as a long compilation and rearrangement of subsequences from preliminary *katas*, especially the so-called *Bassai-dai* and *Heian katas*, which should be well-known by then. Therefore, most intermediate practitioners supposedly possess extensive experience with some or all of the preliminary *katas* but have limited, if any, knowledge of the *Kanku-dai*. Even advanced practitioners might commonly fall prey to memory interference effects due to wrong matching and association of the corresponding movement patterns. This constitutes an interesting and challenging scope of application for analyzing mental representation structures, error prediction and performance assessment. The study focused on the first 17 moves from the beginning of *Kanku-dai* up to the first *manji uke* blocking technique:

1. *Morote age shuto uke* (rising knife hand block with both hands)
2. *Chudan shuto uchi* (inside knife hand strike)
3. *Jodan haishu uke* with left arm (back hand block at head height)
4. *Jodan haishu uke* with right arm (back hand block at head height)
5. *Chudan tate shuto uke* (inside vertical knife hand block)
6. *Chudan choku zuki* with right arm (straight punch at middle level)
7. *Uchi uke* with right arm (forearm block)
8. *Chudan choku zuki* with left arm (straight punch at middle level)
9. *Uchi uke* with left arm (forearm block)
10. *Jodan uraken uchi* + *yoko geri* (back fist strike at head height + side kick)
11. 1st *shuto uke* (left arm) (knife hand block)
12. 2nd *shuto uke* (right arm) (knife hand block)
13. 3rd *shuto uke* (left arm) (knife hand block)
14. *Chudan gohon nukite* (five-finger spear hand strike at middle level)
15. *Age shuto uke* (rising knife hand block)
16. *Jodan mae geri* (front kick at head height)
17. *Manji uke* (swastika-shaped double block).

### 3.1. Statement of Ethical Approval

The study has been approved by the ethics committee of Bielefeld University in written form according to the guidelines of the German Psychological Society (DGPs) and the Association of German Professional Psychologists (BDP). All participants gave informed and written consent to participate in the study.

### 3.2. Participants

Twelve individuals between 18 and 63 years with a mean age of 30.7 years (*SD* = 13.3) participated in the study. The majority (75%) of participants were male. Some basic experience in karate, as indicated by holding at least the sixth *kyu* rank (“green belt”), was required to enable proper determination of individual techniques. This was necessary since the SDA-M-based analyses in this study were concerned with action selection mechanisms for choosing between different karate techniques within the *kata* sequence. The cognitive action architecture model allocates these mechanisms primarily to the level of “mental control” and the associated “basic action concepts” (BACs) as mental representation units (Schack, 2004). The corresponding SDA-M-based analyses in this study were inherently and deliberately indifferent to the quality of individual karate techniques. Therefore, participants had to know and apply these BACs, i.e., execute karate techniques, sufficiently well to allow the experimenter to properly and unambiguously recognize and distinguish them. Table 1 shows the exact distribution of participant numbers across formal ranks of expertise. They were reimbursed for their time with 5 Euros in cash.

**Table 1:** Formal expertise of participants in karate.

**Rank**	**6th *kyu***	**5th**	**4th**	**3rd**	**2nd**	**1st *kyu***	**1st *dan***	**2nd *dan***
No. of participants	2	3	1	1	1	0	1	3

*Note that expertise increases from left to right, because kyu ranks traditionally decrement from eighth (beginner) to first kyu (advanced student), whereas the subsequent dan ranks (”master level”) are counted upwards from 1st dan*.

### 3.3. Procedure

First, participants were welcomed, asked to give informed consent to participation, and provide demographic data, as well as their degree of formal expertise in karate. The following proceedings of each trial could be divided into three consecutive phases:

#### 3.3.1. Recapitulation and Learning Phase

A brief recapitulation of preliminary *katas* served both as a physical warm-up and cognitive trigger for activating relevant memory structures. This included the *Heian Nidan, Heian Yondan*, and *Bassai-dai*, which contain similar or identical parts as *Kanku-dai*, each from beginning until the first occurrence of a *kiai*.^1^ Participants who had already been tested in a given *kata* as part of an official examination for their *kyu* or *dan* grade were merely asked to demonstrate it once, in a calm and serene manner, without further guidance. The remaining preliminary *katas* were at least once roughly synchronously executed by the participant and the experimenter as an instructor. If participants made mistakes or struggled noticeably the execution was repeated up to two times. Afterwards, a video was shown of the *Kanku-dai* sequence performed by Master Hirokazu Kanazawa (10th *dan* black belt; †8 December 2019). Participants were then rudimentarily taught to execute this sequence by following the moves in rough synchrony with the experimenter. The number of repetitions depended on formal expertise ranks: Relative beginners (eighth to fifth *kyu*) executed the sequence twice, advanced students (fourth to first *kyu*) executed it once, and black belts did no physical execution at all. The video of the *Kanku-dai* sequence was then shown a second time to finalize the learning phase.

#### 3.3.2. SDA-M Introduction and Split Procedure

The SDA-M split procedure was explained by showing participants a special tutorial video included in the QSplit software, which specifies the instructions as follows (translated from German to English):

*The software shows representations of two action steps. You shall judge whether these action steps are sequentially “directly associated” during task execution or not, i.e., whether they are executed immediately before or after one another. It does not matter which action step is shown on the left or on the right side of the screen*.

The tutorial video continues to illustrate the implications of these instructions using, as a simple example from daily life, an action sequence for toasting white bread slices and the respective decisions in a corresponding split procedure. Participants were asked to confirm whether they had understood these general instructions. After this, they were subjected to an SDA-M split procedure, which incorporated still images of the first 17 techniques of the *Kanku-dai kata* and corresponding textual descriptions. As usual in karate, Japanese terms were used to denote the techniques. These can be seen in correct order at the bottom of Figure 3. The user interface of QSplit, which was used for this split procedure, is shown in Figure 1.

#### 3.3.3. Movement Sequence Execution Test

Lastly, participants' capability to freely execute the *Kanku-dai* movement sequence was tested. Participants started the *kata* with their back toward the experimenter, so they could not see the experimenter during the movement sequence execution. The experimenter observed the execution and intervened when errors occurred. In this case the experimenter told participants to freeze in their current position, walked in front of them, and demonstrated the correct technique. Participants should then reverse their previous (wrong) action and continue with the correct execution. This intervention procedure was beforehand explained and demonstrated. Importantly, merely slightly inaccurate action executions were ignored as long as the correct technique was still clearly recognizable. Only wrongly chosen techniques were counted as errors and corrected.

### 3.4. Data Analysis

All SDA-M procedures were executed with the *QSplit SDA-M Suite* v1.6 for Windows. This included the split procedure and the usual data normalization, scaling, clustering and invariance analysis steps as described by Schack (2012), as well as advanced analyses using the AMPA and CASPA algorithms (see Strenge et al., 2019). Generally, the available data were analyzed on two different levels:

First, on the level of individual karate techniques, the algorithmic predictions by AMPA, CASPA_*d*_ and CASPA_*i*_ for each action of every participant were compared with the corresponding outcomes during actual execution. For this purpose, several standard metrics for the evaluation of binary classifiers were used. In this context a “true positive” case was counted when the algorithmic analysis predicted an error and this error actually occurred.

Second, participants overall performances, i.e., total numbers of correct actions, and their formal expertise ranks were compared with different SDA-M-based measures, which aim to reflect the overall suitability of individual mental representation structures for the movement task. One of these measures stemmed from Lander and Lange (1992) and Schack (2012), who proposed the structural invariance measure λ. Let the sets *S*_*a*_ and *S*_*b*_ represent the outcomes of SDA-M's hierarchical agglomerative average-linkage clustering for participant *a* and participant *b*, which contain the clusters *C*_*i*_ ∈ *S*_*a*_ and *C*_*j*_ ∈ *S*_*b*_ of BACs (here: karate techniques). The invariance of the mental representation structures of participants *a* and *b* is then defined as follows:

(1) $$\begin{array}{l} \lambda_{a,b}=\sqrt{\frac{\min\left(\left|S_{a}|,|S_{b}\right|\right)}{\max\left(\left|S_{a}|,|S_{b}\right|\right)}\cdot \frac{\sum_{i=1}^{\left|S_{a}\right|}\sum_{j=1}^{\left|S_{b}\right|}|C_{i}\cap C_{j}|}{\sum_{i=1}^{\left|S_{a}\right|}\sum_{j=1}^{\left|S_{b}\right|}\sqrt{\left|C_{i}|\cdot |C_{j}\right|}}};\lambda_{a,b}\in \left[0,1\right] \end{array}$$

More recently, the Adjusted Rand Index (ARI) gained popularity among SDA-M researchers for measuring the similarity of two participants' mental representation structures (see e.g., Frank et al., 2013, 2014, 2016; Land et al., 2014; Jeraj et al., 2017; Kim et al., 2017; Meier et al., 2020). The ARI is bounded above by a maximum of 1 and takes on negative values (with no well-defined lower bound) when similarity falls below the expected value from random clustering with the same number of clusters and elements in each (Hubert and Arabie, 1985). Note that both the invariance measure λ and the ARI are based on SDA-M clustering results. This implies they require a reference structure for comparison, e.g., from one or multiple domain experts. In the present study, an ideal reference structure for this purpose was established by perfectly associating the action representations that exactly precede or follow each other in the movement sequence.

In addition to these two established measures (λ and ARI), CASPA_*m*_ is newly introduced as an advanced alternative. It represents the arithmetic mean over all likelihoods of successful action selection during the whole sequence of movements as predicted for an individual by the CASPA algorithm. Formally, if *n* is the number of actions in the designated action sequence (here: *n* = 17) and *p*_*i*_ the probability of correct action selection for a given participant after executing a previous action *a*_*i*_ as estimated by CASPA, then CASPA_*m*_ is defined as follows:

(2) $${\text{CASPA}}_{m}≔\frac{\text{1}}{n-1}\sum_{i=1}^{n-1}p_{i};{\text{CASPA}}_{m}\in [\text{0},\text{1}]$$

This value can also be interpreted as an overall estimate of the expected probability of correct action selection for a randomly chosen situation within the sequence.^2^ CASPA_*m*_ has the advantage over previous alternatives (the invariance λ and ARI) that it does not require an explicit reference structure. CASPA_*m*_ also inherits a notable limitation of the CASPA algorithm though: It is only applicable to SDA-M data sets related to action sequences that have no temporal overlap between the actions. Therefore, it cannot generally replace λ and ARI for arbitrary SDA-M application scenarios if this condition is not satisfied.

## 4. Results

Substantial, albeit imperfect, matches between algorithmic analyses of participants' mental representation structures and their actual accomplishments while executing the movement sequence were found.

**Figure 5:**
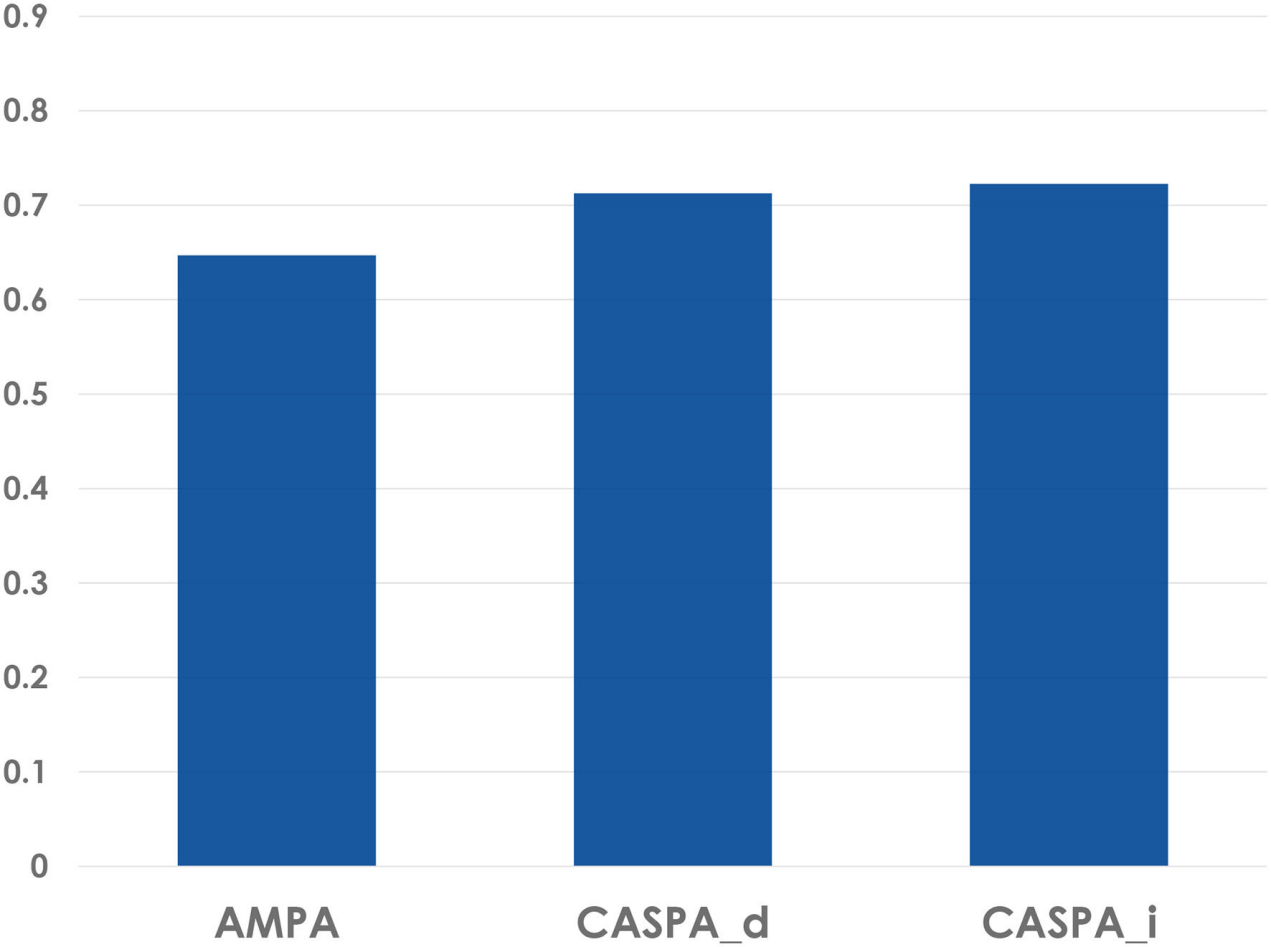
Balanced accuracies of different SDA-M-based algorithms for error prediction in the *Kanku-dai* sequence.

Detailed metrics for the performance of AMPA, CASPA_*d*_ (using the default threshold of 0.5), and CASPA_*i*_ (using an empirically informed threshold of 0.6207), with respect to predicting participants individual likelihood of making mistakes at the level of each individual action (i.e., discrete karate techniques) are shown in Table 2. An overall relatively low prevalence of errors (31 errors in a total of 192 actions ⇒*P*(error) ≈ 16%) caused a salient discrepancy between positive and negative predictive values (PPVs ∈ [0.29, 0.31] and NPVs ∈ [0.90, 0.94]). However, the prevalence-independent measures of sensitivity (values ∈ [0.55, 0.77]) and specificity (values ∈ [0.67, 0.75]) were rather close to each other. From an applied perspective sensitivity matters for recognizing as many of the practitioners weak points as possible, whereas specificity helps focusing on these issues instead of unnecessarily practicing parts they already mastered. The CASPA_*i*_ algorithm achieved the best results among the different algorithmic variants in terms of balanced accuracy (value 0.72; see Figure 5), which represents the arithmetic mean of sensitivity and specificity values (Brodersen et al., 2010).

**Table 2:** Detailed results of SDA-M-based error prediction in the *Kanku-dai* sequence.

**Algorithm**	**Accuracy**	**Sensitivity**	**Specificity**	**PPV**	**NPV**	**Balanced accuracy**
AMPA	0.71^***^	0.55	0.75	0.29	0.90	0.65
CASPA_*d*_	0.69^***^	0.74	0.68	0.31	0.93	0.71
CASPA_*i*_	0.69^***^	0.77	0.67	0.31	0.94	0.72

*** *p ≪ 10^−5^*.

Table 3 shows the correlations (using Spearman's rank-order correlation coefficient ρ) between participants' formal expertise (*kyu*/*dan* rank), their actual performance in the *Kanku-dai kata* execution test (i.e., number of correctly chosen techniques), the conventional SDA-M measures for assessing the invariance and similarity of individual mental representation structures to an ideal reference structure (Lander's λ and ARI)^3^, and the newly proposed CASPA_*m*_ measure. All three SDA-M-based assessment metrics showed significant and strong positive correlations with participants actual performances (CASPA_*m*_:ρ = 0.88, *p* < 0.001; Lander's λ:ρ = 0.79, *p* < 0.01, ARI: ρ = 0.65, *p* < 0.05). CASPA_*m*_ and Lander's λ also correlated significantly and strongly with formal expertise ranks (CASPA_*m*_:ρ = 0.80, *p* < 0.01; Lander's λ:ρ = 0.66, *p* < 0.05). The differences between Lander's λ, ARI, and CASPA_*m*_'s correlations with performance and expertise were statistically insignificant (using Fisher *z*-transformation for comparison of the correlation coefficients). However, CASPA_*m*_ descriptively showed the strongest correlations with performance and expertise among all three SDA-M-based metrics, and also showed higher correlations with actual performance than formal expertise ranks did (CASPA_*m*_:ρ = 0.88 vs. formal expertise: ρ = 0.84).

**Table 3:** Correlations between formal expertise, actual performance, and SDA-M-based assessment metrics.

	**Expertise**	**Performance**	**CASPA_***m***_**	**Invariance λ**
Performance	.84^***^			
CASPA_*m*_	.80^** b)^	.88^***^		
Invariance λ	.66^* a)^	.79^** c)^	.80^** a)^	
ARI	.44	.65^* b)^	.49	.75^** d)^

*** p < 0.001

**a) p = 0.0016

**b) p = 0.0018

**c) p = 0.0022

**d) p = 0.0047

*a) p = 0.02

*b) *p = 0.023*.

On a sidenote, we recognized that the two most error-prone steps occured when transitioning from the 9th action (*uchi uke* block with left arm) to the 10th action (*jodan uraken uchi* back fist strike with *yoko geri* sidekick) with 5/12 errors and from the 16th action (*jodan mae geri* front kick) to the 17th action (*manji uke* block) with 6/12 errors. In both of these cases, the preceding techniques marked the end of corresponding movement sub-sequences known to some participants from preliminary katas, which at the respective point would go on with other techniques than the tested *kanku-dai* kata demands. Participants up to the 3rd *kyu* grade (brown belt) made errors at these points, which corroborates the supposition that memory interference effects may play an important role in learning and distinguishing these katas.

## 5. Discussion and Ethical Considerations

Deliberate practice has generally been accepted as an important factor for developing expertise, especially in sports, even though the specific extent of its impact on performance remains a subject of debate (cf. Ericsson, 2008; Anderson, 2010; Macnamara et al., 2016). By definition, deliberate practice requires that a coach or trainer sets specific individual training goals and provides feedback to practitioners. This may constitute a blocking obstacle when no coach is available, e.g., during travel or exercise at home. Motivated by prior research results and applications of the SDA-M method the present study investigated whether automatized SDA-M-based assessments could serve as an approximate technical substitute for the role that human coaches fulfill in deliberate practice. This included identifying potential issues and assessing a practitioner's overall competency with respect to specific movement sequences to derive feasible training goals.

The present study focused on choosing correct movements, not on improving individual actions' execution quality. Arguably, assisting deliberate practice on the level of basic action selection rather than the level of atomic action features seems especially helpful for intermediate and advanced practitioners, since Ericsson (2008, p. 991) noted that after sufficient practice “*the aspiring expert performers become able to monitor their performance so they can start taking over the evaluative activity of the teacher and coach. They acquire and refine mechanisms that permit increased control, which allow them to monitor performance in representative situations to identify errors as well as improvable aspects.”* While this kind of self-monitoring might work well for recurring basic actions, like well-known karate techniques, it cannot prevent mistakes in insufficiently practiced action sequences.

Albeit preliminary due to a limited sample size, the empiric results are highly promising: SDA-M-based algorithms reached accuracy values that were highly significant above chance level and correctly predicted up to 77% of all actual errors in action selection during the tested karate movement sequence. In deliberate practice, this information could be used by coaches, practitioners, and CIT-based training assistance systems to focus on practicing corresponding subsequences including (at least) the preceding and subsequent techniques surrounding the practitioner's most error-prone action steps in order to strengthen associations between these actions.

Furthermore, SDA-M-based measures for assessing the overall suitability of participants' individual mental representation structures, especially the newly proposed CASPA_*m*_ metric, correlated significantly and strongly with karate practitioners actual performances. After analyzing someone's mental representation structures related to different relevant movement sequences (e.g., a set of several *katas* that may need to be performed in their next belt examination), these metrics could be used to focus deliberate practice on poorly rated sequences, i.e., those that are not yet sufficiently established in practitioners' memory.

A notable limitation of the currently available algorithms for automatized SDA-M-based assessments and error predictions is that they require a predefined, limited set of correct action sequences in terms of basic actions. This makes them potentially applicable not only to martial arts forms and dance choreographies but also to opening sequences in chess or real-time strategy games (B. Strenge et al., unpublished) and other fixed sequences of basic actions that do not overlap in time. However, they cannot readily be applied to more dynamic, impulsive and spontaneous situations in sports and training that do not satisfy these requirements.

A mobile CIT assistance system, e.g., based on smart glasses, could use the information from SDA-M-based analyses to suggest training goals, provide feedback, and track practitioners' learning curves in terms of developing task-related memory structures over time. Such a system would enable intermediate practitioners to engage in deliberate practice of action sequences anywhere anytime instead of requiring personal contact with their coaches. Arguably, this would entail a broad range of ethically relevant aspects:

- Greater independence from organizational structures like sports clubs,
- Less time spent and environmental damage due to regular traveling,
- More flexible training schedules,
- Better opportunities for independent adjustment of repetitions in deliberate practice, and
- Prevention of potential embarrassment due to the observation of ones mistakes by other people.

With respect to the current situation concerning the ongoing COVID-19 pandemic and impending climate catastrophe, one might add that special circumstances make many of these aspects all the more relevant and pressing issues.

**Figure 6:**
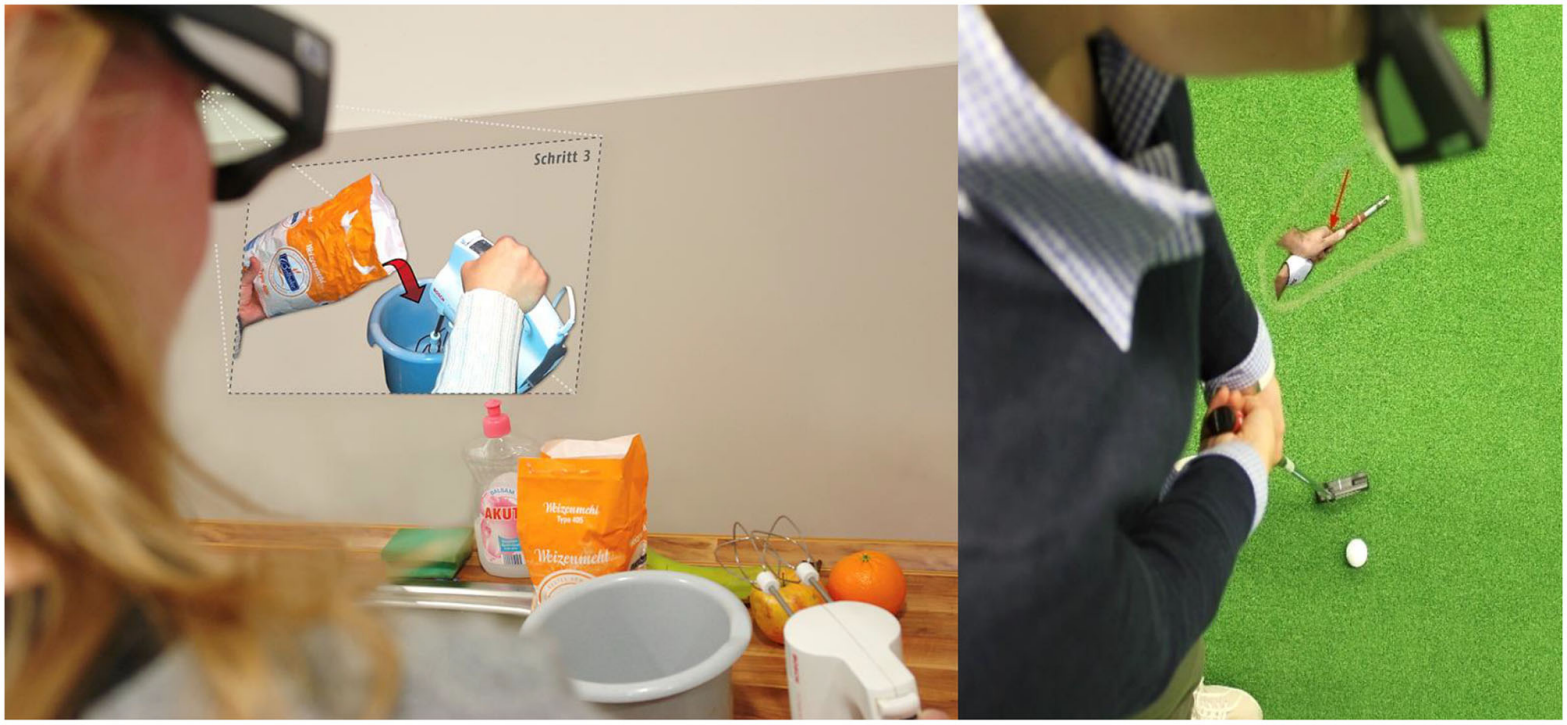
Based on the measurement of mental representation structures, it is possible to learn about the expertise stage of the user and to provide individualized feedback (e.g., in a bakery or golf putting scenario). Photos: CITEC. Reproduced with permission of Thomas Schack.

In another research direction, which could be interesting for anticipation in sport and medicine, researchers tried to support activities by seeing the world through assistive glasses. This project, called ADAMAAS (*Adaptive and Mobile Action Assistance in Daily Living Activities*), focused on the development of a mobile adaptive assistance system in the form of intelligent glasses, which provide unobtrusive, anticipatory, and intuitive support in everyday situations (Essig et al., 2016). The system is able to identify problems in ongoing action processes, react to mistakes, and provide context-related assistance via textual, pictorial, or three-dimensional virtual elements superimposed on a transparent display (see Figure 6). This project investigated the integration of mental representation analysis, eye tracking, physiological measures (e.g., heart rate), computer vision (i.e., object and action recognition), and augmented reality with modern diagnostics and corrective intervention techniques. The major perspectives that distinguish ADAMAAS from stationary diagnostic systems and conventional head-mounted displays include its ability to react to errors in real-time, provide individualized feedback for action support, and learn from the individual behavior of the user. Such intelligent AR glasses could be used to provide athlete- and sport-sensitive feedback, for remote observation or assistance (e.g., transferring the video to the trainer; or the trainer can use a salient pointer to help the athlete to focus on the relevant cue), as well as new forms of training, such as displaying distracting stimuli in the glasses in order to simulate different training conditions or environments (Schack, 2020).

Despite all the new possibilities opened up by the application of new technologies in sport science there are also many challenges that have to be considered: New technologies allow the recording and storage of detailed user-specific data. Therefore, privacy issues and other ethical, legal and social implications (ELSI) are becoming more and more important and are seen as essential considerations with respect to technological developments.

A worthwhile CIT system would need to be developed with the aforementioned and other ethical aspects in mind to ensure that the potential benefits actually come into effect. Therefore, the technical development process should adhere to specific rules regarding the inclusion of ethical issues. This is especially important in contemporary agile development settings that are characterized by transient requirements definitions and short-term prioritization of features. Specialized system design methodologies like “*Value-Sensitive Design”* (Friedman et al., 2008) or the “*Ethical System Design Lifecycle”* (Spiekermann, 2015) define methods and processes for this purpose. In a similar vein, Strenge and Schack (2019) proposed an innovative approach to incorporate ethically relevant criteria during agile development processes through a flexibly applicable methodology called *Agile Worth-Oriented Systems Engineering* (AWOSE). First, a predefined model for the ethical evaluation of sociotechnical systems is used to assess ethical issues according to different dimensions. To ensure that ethical issues are not only identified but also systematically considered during system design, the second part of AWOSE integrates the findings with approaches from worth-centered development into a process model compatible with agile methodologies. Improved artifacts of worth-centered development called Worth Maps guide the prioritization of development tasks as well as choices among design alternatives with respect to ethical implications. Furthermore, the improved Worth Maps facilitate the identification of suitable criteria for system evaluations in association to ethical concerns and desired positive outcomes of system usage.

Future research could focus not only on replicating the current study's findings with more extensive and heterogeneous participant samples and other sample applications but also investigate the long-term applicability and usefulness of the automatized assessment approaches. A major research and development objective could be to build an assistance system and empirically test its impact on the quality and efficacy of deliberate practice compared to unassisted training and/or traditional coach interaction. Finally, a long-term study could verify which (if any) ethically relevant benefits actually arise from using such a system.

## Data Availability Statement

The raw data supporting the conclusions of this article will be made available by the authors, without undue reservation.

## Ethics Statement

The studies involving human participants were reviewed and approved by the ethics committee of Bielefeld University. The patients/participants provided their written informed consent to participate in this study. Written informed consent was obtained from the individual(s) for the publication of any potentially identifiable images or data included in this article.

## Author Contributions

BS was either solely accountable for, or involved in, all aspects of this work. DK contributed to the experimental design and execution, data preparation, and writing. TS provided the theoretical framework for analyzing mental representation structures with SDA-M, supervised the research strategy, and contributed to the writing. All authors contributed to the article and approved the submitted version.

## Conflict of Interest

The authors declare that the research was conducted in the absence of any commercial or financial relationships that could be construed as a potential conflict of interest.

## Footnotes

^1^The *kiai* is a short shout that is uttered when performing distinct moves in karate. The correct execution of *katas* usually requires *kiais* at certain specified points.

^2^This formulation assumes that there is only one correct action sequence to achieve the goal. The case of multiple different correct sequences would require slightly more complex calculations, involving a weighted arithmetic mean that weights the estimated probabilities of correct action selection for each situation with the joint probabilities of having previously chosen exactly the actions needed to get into that situation.

^3^Lander's λ and ARI were both calculated from SDA-M clusterings with a significance level of α = 1 %.

## References

[B1] Anderson J. R. (2010). Cognitive Psychology and Its Implications, 7th Edn. New York, NY: Worth Publication.

[B2] Anderson J. R. Bothell D. Byrne M. D. Douglass S. Lebiere C. Qin Y. (2004). An integrated theory of the mind. Psychol. Rev. 111, 1036–1060. 10.1037/0033-295X.111.4.1036 15482072

[B3] Anderson J. R. Lebiere C. (1998). The Atomic Components of Thought. Mahwah, NJ: Lawrence Erlbaum Associates.

[B4] Brodersen K. H. Ong C. S. Stephan K. E. Buhmann J. M. (2010). The balanced accuracy and its posterior distribution, in 20th International Conference on Pattern Recognition (ICPR) (Istanbul), 2010, 3121–3124. 10.1109/ICPR.2010.764

[B5] Ericsson K. A. (2007). Deliberate practice and the modifiability of body and mind: toward a science of the structure and acquisition of expert and elite performance. Int. J. Sport Psychol.38, 4–34.

[B6] Ericsson K. A. (2008). Deliberate practice and acquisition of expert performance: a general overview. Acad. Emerg. Med. 15, 988–994. 10.1111/j.1553-2712.2008.00227.x 18778378

[B7] Essig K. Strenge B. Schack T. (2016). ADAMAAS: towards smart glasses for mobile and personalized action assistance, in Proceedings of the 9th ACM International Conference on PErvasive Technologies Related to Assistive Environments (PETRA '16), PETRA '16 (New York, NY: ACM), 46:1–46:4. 10.1145/2910674.2910727

[B8] Fitts P. M. Posner M. I. (1967). Human Performance. Belmont, CA: Brooks/Cole.

[B9] Frank C. Land W. M. Popp C. Schack T. (2014). Mental representation and mental practice: Experimental investigation on the functional links between motor memory and motor imagery. PLoS ONE 9:e95175. 10.1371/journal.pone.0095175 24743576 PMC3990621

[B10] Frank C. Land W. M. Schack T. (2013). Mental representation and learning: the influence of practice on the development of mental representation structure in complex action. Psychol. Sport Exerc. 14, 353–361. 10.1016/j.psychsport.2012.12.001

[B11] Frank C. Land W. M. Schack T. (2016). Perceptual-cognitive changes during motor learning: the influence of mental and physical practice on mental representation, gaze behavior, and performance of a complex action. Front. Psychol. 6:1981. 10.3389/fpsyg.2015.01981 26779089 PMC4705276

[B12] Friedman B. Kahn P. H. Borning A. (2008). Value sensitive design and information systems, in The Handbook of Information and Computer Ethics, eds Himma K. E. Tavani H. T. (Hoboken, NJ: John Wiley & Sons, Inc.), 69–101. 10.1002/9780470281819.ch4

[B13] Hagan J. E. Jr. Schack T. Koester D. (2018). Passion play. Embracing new scientific perspectives for improved sport psychology consulting. SOJ Psychol. 4, 1–5. 10.15226/2374-6874/5/1/00143

[B14] Heinen T. Schack T. (2004). Bewegungsgedächtnis und Bewegungsausführung-Optimierung von Rotationsbewegungen im Gerätturnen. Lehren und Lernen im Turnen. Veröffentlichungsband zur Jahrestagung, 85–95.

[B15] Heinen T. Schwaiger J. (2002). Optimierung des Trainingsprozesses im Kunstturnen durch kognitive Verfahren, in Expertise im Sport: Lehren, Lernen, Leisten, eds Strauß B. Tietjens M. Hagemann N. Stachelhaus A. (Cologne: bps) 67–68.

[B16] Heinen T. Schwaiger J. Schack T. (2002). Optimising gymnastics training with cognitive methods, in Proceedings of 7th annual Congress of the European College of Sport Science (Athens), 608.

[B17] Hodges N. J. Huys R. Starkes J. L. (2007). Methodological review and evaluation of research in expert performance in sport, in Handbook of Sport Psychology, eds Tenenbaum G. Eklund R. C. (Hoboken, NJ: John Wiley & Sons), 161–183. 10.1002/9781118270011.ch7

[B18] Hossner E.-J. Schiebl F. Göhner U. (2015). A functional approach to movement analysis and error identification in sports and physical education. Front. Psychol. 6:1339. 10.3389/fpsyg.2015.01339 26441717 PMC4564696

[B19] Hubert L. Arabie P. (1985). Comparing partitions. J. Classif. 2, 193–218. 10.1007/BF01908075

[B20] Jeraj D. Musculus L. Lobinger B. (2017). Body image and mental representation in table tennis players who do versus do not use a prosthesis. Probl. Psychol. 21 Cent. 11, 22–30.

[B21] Kim T. Frank C. Schack T. (2017). A systematic investigation of the effect of action observation training and motor imagery training on the development of mental representation structure and skill performance. Front. Hum. Neurosci. 11:499 10.3389/fnhum.2017.00499 29089881 PMC5650990

[B22] Land W. Frank C. Schack T. (2014). The influence of attentional focus on the development of skill representation in a complex action. Psychol. Sport Exerc. 15, 30–38. 10.1016/j.psychsport.2013.09.006

[B23] Lander H.-J. Lange K. (1992). Eine differentialpsychologische Analyse begrifflich-strukturierten Wissens. Zeitschrift für Psychologie mit Zeitschrift für angewandte Psychologie 200, 181–197.

[B24] Macnamara B. N. Moreau D. Hambrick D. Z. (2016). The relationship between deliberate practice and performance in sports: a meta-analysis. Perspect. Psychol. Sci. 11, 333–350. 10.1177/1745691616635591 27217246

[B25] Meier C. Frank C. Gröben B. Schack T. (2020). Verbal instructions and motor learning: how analogy and explicit instructions influence the development of mental representations and tennis serve performance. Front. Psychol. 11:2. 10.3389/fpsyg.2020.00002 32116881 PMC7019697

[B26] Ritter H. (2010). Cognitive interaction technology. Künstl. Intell. 24, 319–322. 10.1007/s13218-010-0063-x

[B27] Ritter H. Sagerer G. (2009). Excellence cluster “cognitive interaction technology”-cognition as a basis for natural interaction with technical systems. Inform. Technol. 51, 112–118. 10.1524/itit.2009.0532

[B28] Schack T. (2004). The cognitive architecture of complex movement. Int. J. Sport Exerc. Psychol. 2, 403–438. 10.1080/1612197X.2004.9671753

[B29] Schack T. (2012). Measuring mental representations, in Measurement in Sport and Exercise Psychology, eds Tenenbaum G. Eklund R. C. Kamata A. (Champaign, IL: Human Kinetics), 203–214. 10.5040/9781492596332.ch-019

[B30] Schack T. (2020). Mental representation in action, in Handbook of Sport Psychology, eds Tenenbaum G. Eklund R. C. (Hoboken, NJ: John Wiley & Sons, Ltd.), 513–534. 10.1002/9781119568124.ch24

[B31] Schack T. Bertollo M. Koester D. Maycock J. Essig K. (2014a). Technological advancements in sport psychology, in Companion to Sport and Exercise Psychology: Global Perspectives and Fundamental Concepts, eds Papaioannou A. G. Hackfort D. (Routledge), 953–965.

[B32] Schack T. Essig K. Frank C. Koester D. (2014b). Mental representation and motor imagery training. Front. Hum. Neurosci. 8:328. 10.3389/fnhum.2014.00328 24904368 PMC4033090

[B33] Schack T. Hackfort D. (2007). An action theory approach to applied sport psychology. Handb. Sport Psychol. 3, 332–351. 10.1002/9781118270011.ch15

[B34] Schack T. Hagan J. E. Jr. Essig K. (2020). New technologies in sport psychology practice, in The Routledge International Encyclopedia of Sport and Exercise Psychology. Volume 2: Applied and Practical Measures, eds Hackfort D. Schinke R. J. (New York, NY: Routledge), 14.

[B35] Schack T. Mechsner F. (2006). Representation of motor skills in human long-term memory. Neurosci. Lett. 391, 77–81. 10.1016/j.neulet.2005.10.009 16266782

[B36] Schack T. Ritter H. (2013). Representation and learning in motor action-bridges between experimental research and cognitive robotics. N. Ideas Psychol. 31, 258–269. 10.1016/j.newideapsych.2013.04.003

[B37] Spiekermann S. (2015). Ethical IT Innovation. Boca Raton, FL: Auerbach Publications 10.1201/b19060

[B38] Strenge B. Schack T. (2019). Awose – A process model for incorporating ethical analyses in agile systems engineering. Sci. Eng. Ethics 26, 851–870. 10.1007/s11948-019-00133-z 31588964 PMC7089881

[B39] Strenge B. Vogel L. Schack T. (2019). Computational assessment of long-term memory structures from SDA-M related to action sequences. PLoS ONE 14:e0212414. 10.1371/journal.pone.0212414 30794606 PMC6386273

[B40] Tenenbaum G. Hatfield B. D. Eklund R. C. Land W. M. Calmeiro L. Razon S. (2009). A conceptual framework for studying emotions-cognitions-performance linkage under conditions that vary in perceived pressure, in Mind and Motion: The Bidirectional Link Between Thought and Action, Vol. 174, eds Raab M. Johnson J. G. Heekeren H. R. (Elsevier), 159–178. 10.1016/S0079-6123(09)01314-4 19477338

[B41] Wachsmuth I. (2008). Cognitive interaction technology: humans, robots, and max, in International Conference on Informatics Education and Research for Knowledge-Circulating Society (ICKS 2008) (Kyoto), 4–5. 10.1109/ICKS.2008.34

[B42] Wachsmuth S. Schulz S. Lier F. Siepmann F. Lütkebohle I. (2012). The robot head “Flobi”: a research platform for cognitive interaction technology, in German Conference on Artificial Intelligence, ed Wöfl S. (Saarbrücken: Deutsches Forschungszentrum für Künstliche Intelligenz), 3–7.

[B43] Wrede S. Leichsenring C. Holthaus P. Hermann T. Wachsmuth S. Team T. C. (2017). The cognitive service robotics apartment: a versatile environment for human-machine interaction research. Künstl. Intell. 31, 299–304. 10.1007/s13218-017-0492-x

